# Causes and Consequences of Snake Venom Variation

**DOI:** 10.1016/j.tips.2020.05.006

**Published:** 2020-06-19

**Authors:** Nicholas R. Casewell, Timothy N.W. Jackson, Andreas H. Laustsen, Kartik Sunaga

**Affiliations:** 1 Centre for Snakebite Research and Interventions, Liverpool School of Tropical Medicine, Pembroke Place, Liverpool, L3 5QA, UK; 2 Australian Venom Research Unit, Department of Pharmacology and Therapeutics, University of Melbourne, Victoria, Australia; 3 Department of Biotechnology and Biomedicine, Technical University of Denmark, DK-2800 Kongens Lyngby, Denmark; 4 Evolutionary Venomics Laboratory, Centre for Ecological Sciences, Indian Institute of Science, Bangalore 560012, Karnataka, India

## Abstract

Snake venoms are mixtures of toxins that vary extensively between and within snake species. This variability has serious consequences for the management of the world’s 1.8 million annual snakebite victims. Advances in ‘omic’ technologies have empowered toxinologists to comprehensively characterize snake venom compositions, unravel the molecular mechanisms that underpin venom variation, and elucidate the ensuing functional consequences. In this review, we describe how such mechanistic processes have resulted in suites of toxin isoforms that cause diverse pathologies in human snakebite victims and we detail how variation in venom composition can result in treatment failure. Finally, we outline current therapeutic approaches designed to circumvent venom variation and deliver next-generation treatments for the world’s most lethal neglected tropical disease.

## Snake Venom and Snakebite

Venom is a remarkable evolutionary innovation found scattered across the animal tree of life [1]. Due to their diverse evolutionary histories and consequent variability, animal venoms have proven to be fascinating models for understanding a number of fundamental processes, including gene duplication, genotype-phenotype mapping, convergent evolution, and cell and tissue development [2–6], while the bioactivities of many toxins make them promising leads for the discovery of new human therapeutics [7]. The most well-studied venom systems are those of snakes. All ‘advanced snakes’ (superfamily: Colubroidea) have a pair of homologous oral venom glands located behind the eye on either side of the upper jaw [8,9]. These glands are connected to ducts that transfer the secreted venom to the base of morphologically diverse teeth that are often referred to as ‘fangs’. For many snakes, including those of greatest medical importance, these fangs are found at the front of the mouth, contain an enclosed venom canal, and are a highly efficient mechanism that facilitates the rapid injection of a bolus of venom. Although venomous snakes predominantly use their venom to assist with the acquisition of prey, they may also deploy it in defensive bites to deter potential predators and aggressors, including people.

The consequences of such human snakebites can be severe. Current estimates suggest that venomous snakes cause up to 138 000 deaths worldwide each year and perhaps as many as 500 000 additional cases of venom-induced morbidity [10]. Snakebite envenomings predominantly affect the rural impoverished populations of the tropics and consequently the World Health Organization (WHO) has listed snakebite as a priority **neglected tropical disease (NTD)** (see Glossary)[11]. The pathological effects of snakebite are diverse and can include neuromuscular paralysis (**neurotoxicity**), hemorrhage and coagulopathy (**hemotoxicity**), and/or local swelling, blistering, and tissue necrosis (**cytotoxicity**) around the bite site [10]. These highly variable clinical signs are a direct consequence of variation in the toxin components found in venom; such variation can be extensive and occurs both inter- and intraspecifically [12–18].This variation also has a direct impact on the efficacy of snakebite treatments (antivenom), resulting in different antivenoms having to be manufactured against the venoms of distinct snake species [19].

Despite such complexity, recent advances in ‘omic’ technologies (e.g., proteomics, transcriptomics) have enabled the rapid characterization of the toxin components found in the venom of over 125 medically relevant species [20]. Here, we outline how this data has transformed our understanding of the processes that have generated snake **venom variation** and the consequences of such variation in the context of snakebite pathology and treatment. We also highlight how the rational application of venom composition data will enable the development of broadly effective therapies for snakebite envenoming, which is an essential step in mitigating the devastating effects this NTD inflicts upon the vulnerable victims of the tropics [21].

## Ecology Drives Inter- and Intraspecific Snake Venom Variation

Venom is a functional trait used by one organism to interfere with the homeostatic processes of another, generally to facilitate feeding or deter predators or competitors [18]. Venom is therefore intrinsically ecological; a trait that mediates the outcome of interactions between two or more organisms [22]. ‘Venomous’ is not synonymous with ‘dangerous’, and the majority of venomous organisms, including snakes, pose no threat to humans, either because they rarely, if ever, envenom humans, or because the consequences of envenoming are trivial. Indeed, venom is a widespread trait amongst the ‘advanced snakes’, but almost all ‘medically important’ snakes (those capable of causing harm to humans via envenoming) are members of one of only three clades: the families **Elapidae** (cobras, mambas, sea snakes, taipans, and their relatives) and **Viperidae** (vipers and pit vipers, including adders, rattlesnakes, and their relatives) and the subfamily Atractaspidinae (mole vipers/stiletto snakes).

As venom is an ecologically important functional trait for venomous snakes, its composition and activity coevolves with the physiology of the prey animals and, perhaps to a certain extent, the predators it is deployed against [23–27]. Although primates have been predators of snakes since time immemorial and envenoming of humans by snakes is almost exclusively defensive, it is unlikely that humans have exerted any major defensive selective pressure on snake venoms. Rather, human envenomings are best viewed as collateral damage of the chemical arms race taking place between venomous snakes and their (mammalian) prey.

Venom variation exists at multiple phylogenetic levels and is a consequence of both the contingent evolutionary histories of divergent lineages of venomous snakes and direct selection on the ecological deployment of specific toxins. At the deepest and most general level, the venoms of, for example, elapid and viperid snakes are different(Box 1);certain families of toxins have been recruited and utilized or have become central components of the venom of one lineage but not the other [28]. Similarly, broad differences can exist in venom compositions between genera within each family and between species within each genus (e.g., [13,20,29]). This much has long been understood and is why a number of antivenom manufacturers have developed multiple products for use in a given region (e.g., viper- and elapid-specific antivenoms).

More recently, the extent of venom variation within species has begun to be recognized. Such variation exists between populations (i.e., regional variation) and between age/size classes [12,14–17]. As venom is a dynamically evolving ecological trait, it stands to reason that whenever groups differ in their feeding ecology, there may be a corresponding difference in their venom composition. Juvenile snakes often consume different prey from adults of the same species and may also exhibit different foraging strategies and prey-handling behavior (e.g., juveniles may be nocturnal, whereas adults are more diurnal; juveniles may employ a bite-and-hold strategy, whereas adults may ‘bite and release’, etc.). The dynamism of venom evolution, which has been documented at the molecular level (see later), is further evidenced by the existence of regional variation, which may be linked both to ecological variance amongst populations and to **neutral evolution,** which may be pervasive in venom systems and work in tandem with **positive selection** [30]. This dynamism itself generates another prediction based on evolutionary first principles: for a trait to evolve rapidly, there must be considerable heritable diversity within populations [31]. This prediction of variation in venom amongst adult members of a single population is only beginning to be investigated, but preliminary evidence suggests it will likely be confirmed [32,33].

## The Processes That Underpin Venom Variation

**Figure 1:**
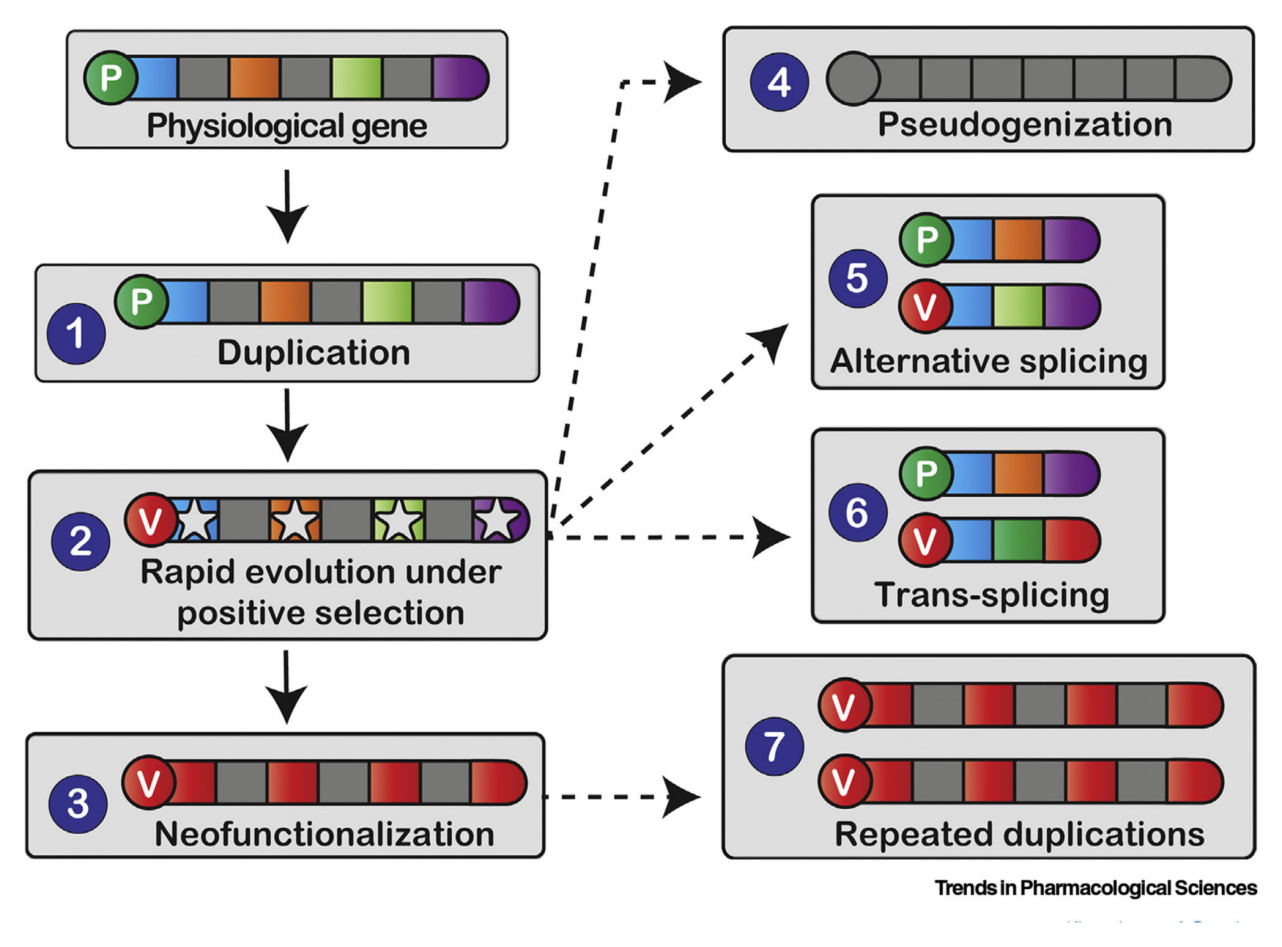
The Molecular and Evolutionary Mechanisms That Underpin the Origin and Diversification ofSnake Venom Toxins. This figure depicts various evolutionary mechanisms that underpin the origin and diversification of snake venom coding genes. Here, introns are shown in grey, while exons are depicted in various colors. Following their origin from (endo)physiological homologues (P) via (1) duplication, snake venom coding genes (V) rapidly accumulate variation under the influence of (2) positive Darwinian selection. On rare occasions, this process results in (3) the origin of novel functions, while it more commonly leads to (4) pseudogenization/degeneration. Snake venom diversity can also be generated via (5) alternative- and (6) trans-splicing, while increased expression can be achieved through (7) repeated gene duplications.

Venom toxin encoding genes originate from genes that code for endophysiological proteins (e.g., salivary, immunological, and pancreatic proteins, etc.) [34]. Numerous mechanisms have been proposed to explain the origin and diversification of toxins and, thereby, the evolution of venom variability across snakes. These include gene duplication, domain loss, evolutionary tinkering of expression levels, alternative- and trans-splicing, and rapid evolution under positive Darwinian selection [35] (Figure 1).

Gene duplication, which plays a key role in the evolution of phenotypic complexity and functional innovation, has been implicated in the diversification of venom. Venom protein encoding genes are theorized to undergo extensive duplications and evolve under a ‘birth and death’ model of evolution [36]. According to this model, repeated duplication events lead to the origin of new copies, most of which undergo pseudogenization into dysfunctional forms over time, while some subsequently evolve novel functions and are retained [37]. Although some assumptions of the original ‘birth and death’ model are questionable [38], gene duplication has led to the formation of multi-locus toxin gene families with extraordinary structural and functional diversity [2,29,39] (see later). Gene duplication does not always introduce novelty though and can also underpin increased expression levels. Concerted evolution, by contrast to the ‘birth and death’ model, maintains high levels of sequence conservation amongst duplicates through recombination, as a strategy to increaseexpression levels of the encoded toxin [40]. Although concerted evolution is yet to be noted in snakes, recurrent snake venom gene duplications do facilitate increased expression of the encoded toxin types [41]. Since relative differences in the expression level of venom components can considerably alter the underlying toxicity [16], shifts in gene expression seem likely to also underpin evolutionary adaptations.

Stochastic degeneration of genes has also been reported to result in significant evolutionary consequences [42]. For example, several lineages of rattlesnakes have lost phospholipase A_2_ (PLA_2_) neurotoxin genes, which were once present in their common ancestor, and have shifted towards a more hemotoxic venom profile [2]. Partial degeneration of gene segments have also been documented in venom protein encoding genes. Domain loss has been shown to mediate toxin **neofunctionalization** (Figure 1), with notable examples including truncations of snake venom metalloproteinases (SVMP) that led to the origin of structurally functionally diverse subclasses in Viperidae snakes [43,44] and the evolution of potent neurotoxins from hemorrhagic precursors in the olive whip snake (*Psammophis mossambicus*) [45]. Recent studies have also highlighted the role of alternative- and trans-splicing in generating snake venom diversity (Figure 1). Genome-wide surveys of the transcriptional repertoire have led to the identification of alternativesplicing in genes that encode SVMPs, snakevenom serine proteases (SVSP) and vascular endothelial growth factors (VEGF), and trans-splicing in SVSP genes [42,46].

Rapid evolution under positive selection has been widely documented to facilitate adaptations in the natural world. Many toxin-encoding genes are known to rapidly accumulate **nonsynonymous substitutions** in their protein encoding regions, relative to **synonymous substitutions.** Three-finger toxins (3FTx), which are amongst the most gene-rich of the toxin superfamilies found in snake venoms, have predominantly evolved under the influence of positive selection, and this process has generated remarkable structural and functional diversity (see later). Similarly, many other toxin classes, including SVMP, PLA_2_, and cysteine-rich secretory proteins (CRISP) have experienced a significant influence of positive selection [43,47–49].

While venom protein superfamilies are generally characterized by extreme conservation of structural residues, particularly disulfide bridge-forming cysteines that confer structural stability, they accumulate variations in other regions [50]. The acquisition of variation in surface-exposed regions and loops, for example, is known to facilitate the rapid diversification and neofunctionalization of toxins [51,52]. Over evolutionary time, venom proteins accumulate variations in an episodic fashion. While **purifying selection** governs the conservation of structur-ally and functionally important residues in potent toxins, positive selection accelerates the rate of change mostly when significant shifts in ecology and environment are experienced [53]. In summary, although a number of mechanisms may contribute, the processes of gene duplication and positive selection appear to be the predominant mechanisms for generating diversity in snake venoms.

## Functional Consequences of Venom Variation

**Figure 2:**
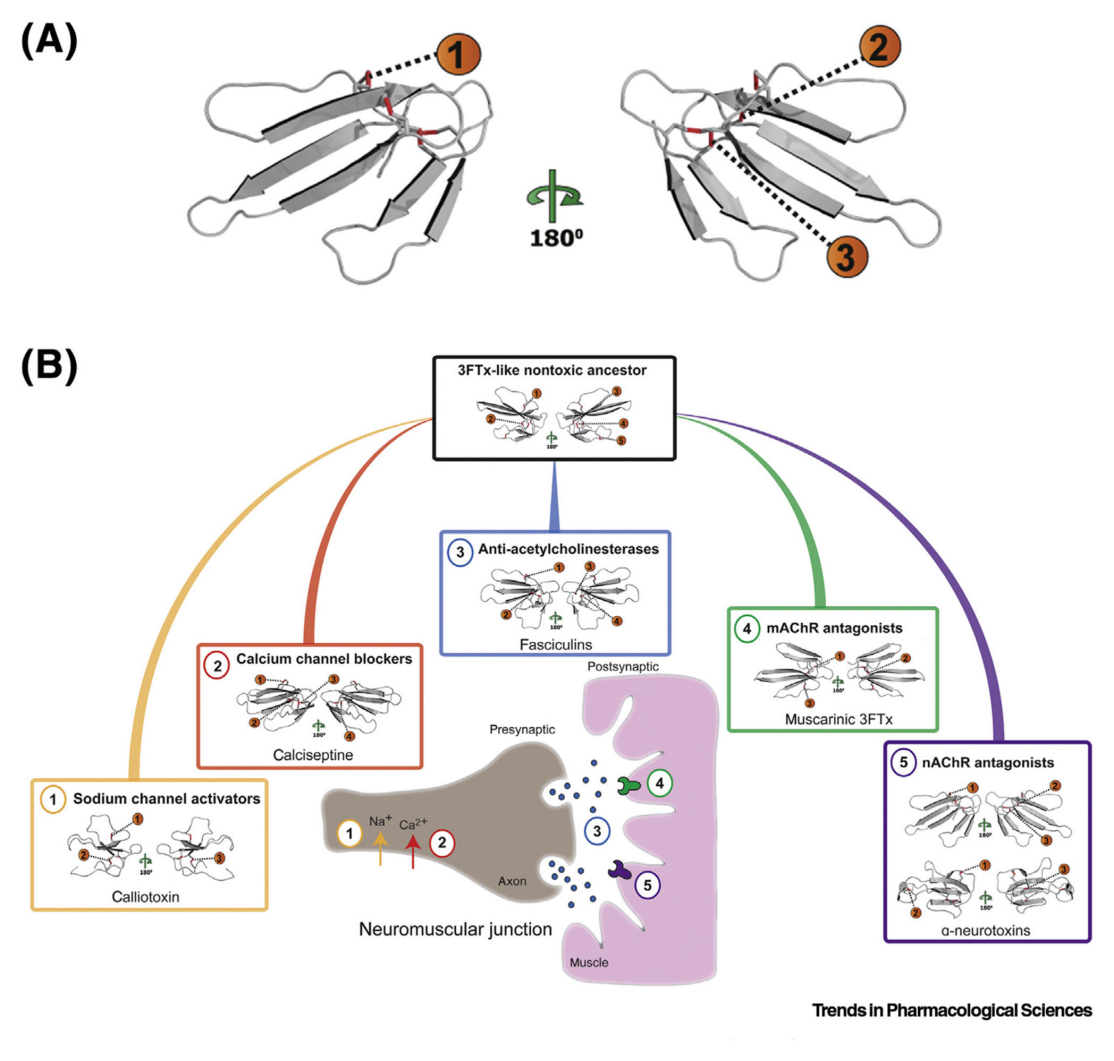
The Structural and Functional Diversity of Three-Finger Toxins (3FTxs). (A) Structural model for a ‘typical’ 3FTx, the short-chain -neurotoxin cobrotoxin (Protein Data Bank: 1COE, from *Naja* *atra*), highlighting the multiple β-hairpin loops extending from the disulfide bond-stabilized hydrophobic core. Disulfide bond numbers are colored red. (B) Via the processes of gene duplication and positive selection, 3FTxs have diversified from a plesiotypic form found in basal henophidian snakes (boas and pythons) into a paralogous suite of functionally diverse toxins in ‘advanced snakes’, many of which act on sites at the neuromuscular junction to cause neuromuscular paralysis. Homology models for various subclasses of 3FTx are displayed, with their variable disulfide bond numbers colored red, and their differential sites of action at the neuromuscular junction shown. (1) Calliotoxin activates the voltage-gated sodium channel, Nav1.4; (2) calciseptine selectively blocks L-type calcium channels; (3) fasciculins exert inhibitory activities against acetylcholinesterase; (4) muscarinic 3FTxs antagonize muscarinic acetylcholine receptors (mAChR); (5) both short-chain (top) and long-chain (bottom) -neurotoxins antagonize a variety of different nicotinic acetylcholine receptor (nAChR) subtypes. Note that there are a number of other, functionally distinct, 3FTxs that are not shown here (e.g., cytotoxins, anticoagulant 3FTxs, etc.).

As a result of the various processes described earlier, most snake venoms contain high numbers of related toxin isoforms. Typically, the toxins encoded by such **multilocus gene families** are the most abundant of those found in venom and examples include the 3FTxs, PLA2s, SVMPs, and SVSPs [20]. Notably, all of these toxin families exhibit evidence of multifunctionality [54]. The 3FTxs, which are dominant venom proteins in most elapid snake venoms, are a classic example of this. Here, gene duplication, coupled with accelerated evolution, has resulted in a suite of toxin isoforms, which share a structure consisting of multiple β-hairpin loops extending from a disulfide bond-stabilized hydrophobic core, but which also exhibit considerable variation in the protruding exposed loops that interact with target-site receptors (Figure 2A) [52]. Many 3FTxs exert neurotoxic effects by interacting with ion channel receptors, including nicotinic acetylcholine receptors, muscarinic receptors, potassium channels, calcium channels, and sodium channels (Figure 2B) [54]. The combined action of different 3FTxs found in the same venom likely results in additive or synergistic antagonizing effects and can cause neuromuscular paralysis and respiratory failure in envenomed snakebite victims [10]. However, other 3FTxs have dramatically distinct functional activities, including those that contribute to local tissue damage via direct cytotoxic effects, orthose that interact with hemostatic components, such as Factor X and platelets [54].

Other venom toxin families exhibit similarly high degrees of functional diversity. These include diverse SVMP isoforms that act in concert to induce hemorrhage via the destruction of basement membrane components, while others cause coagulopathy via direct activation or cleavage of blood clotting factors [55]. Many other toxin families also contribute to systemic pathologies by acting synergistically on relevant physiological targets, such as certain PLA_2_ toxins that antagonize presynaptic potassium channels, or SVSPs that activate Factor V or degrade fibrinogen [54,55].

Crucially, the presence, absence, and relative abundances of the numerous different toxin iso-forms found in venom is highly variable across snake species. Thus, not every venom will have every functionally diverse isoform from each toxin family. However, due to variable lineagespecific processes (e.g., gene duplication and loss, rates of evolution, expression levelvariations, etc.), each species harbors its own mixture of toxins. Consequently, the ensuing pathologies observed following human snakebites are also highly variable. These can range from the predominant systemic neurotoxicity observed following bites by many elapid snakes (e.g., kraits, *Bungarus* spp., mambas, *Dendroaspis* spp.), to particularly complex multipathological envenomings following bites by certain viperid snakes, like Russell’s vipers (*Daboia* spp.) [10,56] Perhaps the most extreme clinical examples of intraspecific venom variation are bites by the Mojave rattlesnake (*Crotalus scutulatus*) in the Southwestern USA, which result in considerably different pathologies (e.g., hemotoxic versus neurotoxic) at different ends of a cline covering only tens of miles [12,57].

## Therapeutic Consequences of Venom Variation

Such functional variation makes snake venoms challenging drug targets and has major consequences for the efficacy of snakebite treatments. Antivenoms are made by hyperimmunizing animals (typically equines or ovines) over prolonged periods of time with venom from a number of snake species found in a particular geographical region, before purifying the resulting antibodies (immunoglobulin G or fragments thereof) and formulating them for intravenous delivery to snakebite victims [10]. Consequently, the specificity and efficacy of these therapeutics are inherently linked to those venoms used for immunization and toxin variation results in reduced recognition and neutralization of toxins from different venoms[19]. In addition, different toxin classes have different levels of antigenicity, with low-molecular weight toxins generally being considered less immunogenic than their high-molecular weight counterparts. This can lead to suboptimal antibody responses in the production animal and, therefore, limited efficacy of antivenoms against toxic, but nonimmunogenic venom components [58,59].

Despite these issues, there are some examples where antivenoms appear to exhibit crossneutralizing capabilities against distantly related snake venoms, particularly where species have broadly similar venom compositions as the result of shared ancestry [60], or coincidently as the result of convergent evolution of venom compositions[29]. However, venom variability often undermines cross-species efficacy and can result in grave clinical consequences (Box 2). In subSaharan Africa, antivenom manufactured against the Indian saw-scaled viper (*Echis carinatus*) was used for treating bites by the congeneric West African saw-scaled viper (*Echis ocellatus*). Due to variation in toxin constituents among saw-scaled vipers [13], these antivenoms proved to be highly ineffective, which resulted in case fatality rates increasing from b2% with species-appropriate antivenom to 10-12% [61,62]. In South Asia, antivenom manufactured using Indian Russell’s viper (*Daboia russelii*) venom exhibits low neutralizing potencies against venom from Bangladeshi populations of the same species, suggesting that perhaps five to ten times the normal treatment dose might be needed for effective treatment [15]. Contrastingly, antivenom made in Thailand against the congeneric species *Daboia siamensis* appears to exhibit considerable cross-recognition of venoms from the same species found in distinct geographical locales [63]. In combination, these observations suggest that venom variation makes predictions of antivenom efficacy extremely problematic, although the application of ‘antivenomic’ approaches that quantify the depletion of chromatographically separated and mass spectrometrically identified venom toxins by antivenoms are gaining traction as a predictive technology to help address these challenges [64]. While such venom compositional data can undoubtedly helpto rationally inform appropriate antivenom use, a severe lack of standardized efficacy data at both the preclinical and clinical level [65,66] currently undermines the development of a robust framework for predicting cross-species antivenom efficacy.

Ultimately, venom variation necessitates the manufacture of many different antivenoms worldwide, each with a restricted geographical focus. This has led to a fragmented, largely unsustainable, market that has resulted in the commercial withdrawal and restricted availability of many antivenoms [67], despite these therapeutics being categorized as essential medicines by the WHO. There is therefore an urgent, compelling need to design new snakebite therapeutics capable of circumventing the limitations associated with snake venom variation.

## Can Novel Snakebite Therapeutics Circumvent Venom Variation?

Due to their animal origin, conventional antivenoms have many limitations, including undefined product compositions, batch-to-batch variation, a propensity to elicit adverse reactions in recipients, and typically limited cross-species efficacies due to venom variation [10,68,69]. However, the recent and widespread utilization of ‘omic’ technologies has enabled antivenom researchers to better understand the composition and variability of snake venoms, which in turn has better informed the identification of toxins requiring neutralization [70,71].

**Next-generation antivenoms** currently under development encapsulate a range of different modalities, including monoclonal antibodies and antibody fragments, **nanobodies,** small molecule inhibitors, **aptamers** and **peptides,** metal ion chelators, and antivenoms manufactured using synthetic immunogens [72]. While the latter products are not fundamentally different from conventional antivenoms, as they are still derived from animal polyclonal antibodies [73], the other modalities are entirely different in their composition and manufacture. Although the manufacture of such next-generation antivenoms is not, in itself, dependent on venoms, antivenom formulation and dosing are highly dependent on knowledge of venom composition and toxicity for the indicated snake species.

This necessitates systematic research in snake genomics, (venom gland) transcriptomics, and (venom) proteomics, coupled with informative analyses of which toxins are of greatest pathological relevance. A successful example of this interdisciplinary approach was the demonstration that immunizing horses with a recombinantly expressed short-chain -neurotoxin, designed as the consensus sequence of important 3FTx isoforms found in different elapid snake venoms, resulted in an experimental antivenom with broad in vivo neutralizing capability against the neurotoxic effects of venoms from distinct elapid snake species [74].

Researchers are also using knowledge of venom composition to rationally select oligoclonal or monoclonal antibodies (or fragments thereof) as potential new snakebite therapeutics. For example, it was recently demonstrated that oligoclonal mixtures of recombinant immunoglobulin G antibodies could be used to neutralize the dendrotoxin-mediated in vivo neurotoxicity of black mamba *(Dendroaspis polylepis)* venom [75]. Crucially, this antibody mixture was rationally designed based on prior proteomic and toxicity assessments of the venom [76], illustrating the importance of in-depth knowledge of venom composition in the development of future **recombinant antivenoms.**

In an attempt to circumvent venom variation by providing generic inhibition of specific toxin classes, researchers have also explored the utility of using small molecules as toxin inhibitors, with some notable successes against SVMP and PLA_2_ toxins. For example, it was recently reported that the metal ion chelator and licensed medicine 2,3-dimercapto-1-propanesulfonic acid (DMPS) provides in vivo protection against the local and systemic effects of the SVMP-rich venoms of saw-scaled vipers (*Echis* spp.) [77]. Researchers have also demonstrated the utility of the Phase II-approved peptidomimetic small molecule SVMP inhibitors, batimastat and marimastat, which have been shown to broadly neutralize multiple viperid SVMPs both in vitro and *in vivo* [78–80]. Moreover, several recent studies have demonstrated the highly promising utility of a repurposed Phase II-approved PLA2 inhibitor, varespladib, as a future snakebite therapeutic, as this molecule has been demonstrated to broadly neutralize PLA_2_-mediated pathologies caused by multiple different elapid and viperid venoms [81,82]. Finally, it was recently described that a therapeutic combination of the SVMP inhibitor marimastat and the PLA_2_ inhibitor varespladib provide broad preclinical efficacy against lethality caused by a range of geographically diverse viper venoms [80].

**Figure 3:**
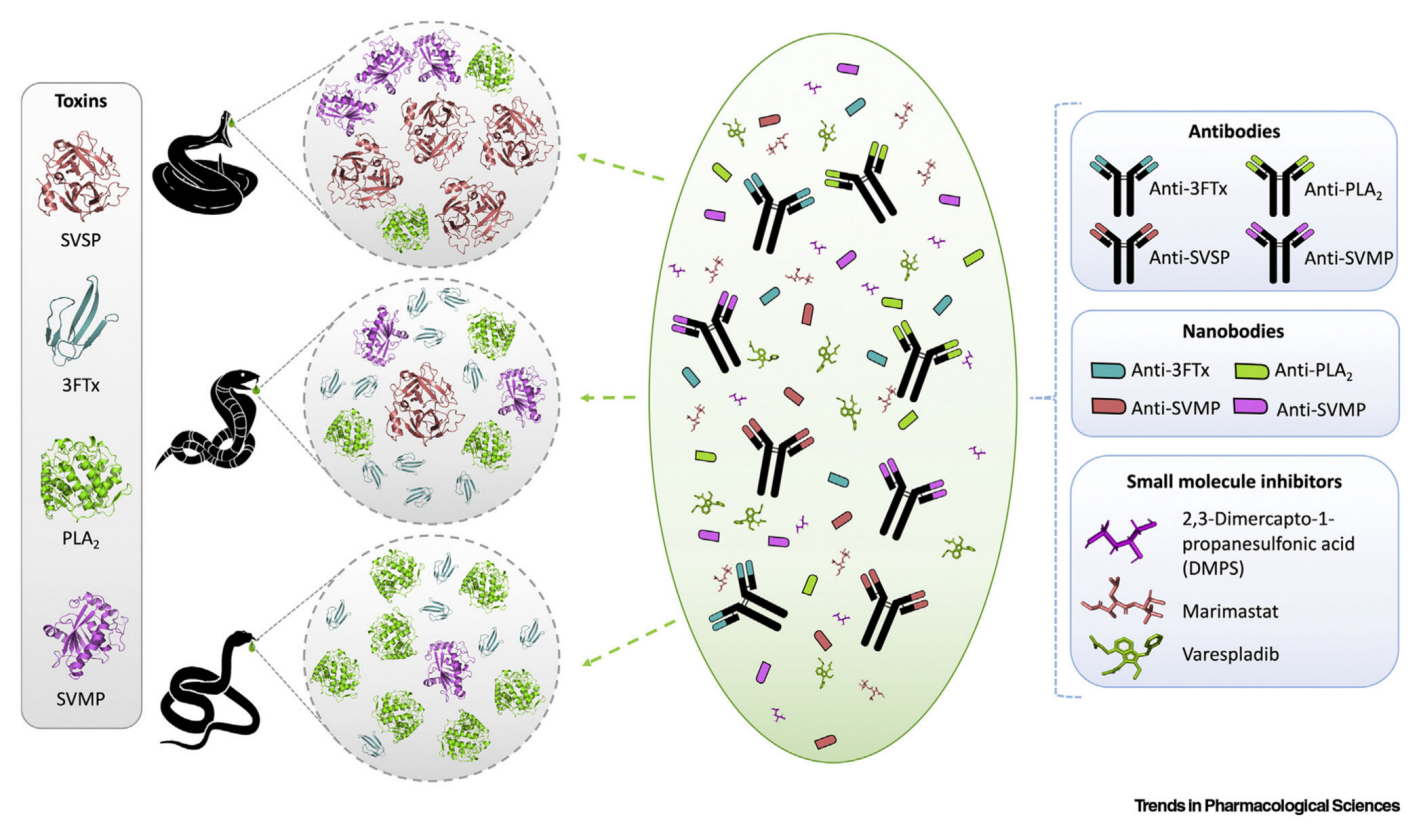
Conceptual Representation of Next-Generation Antivenoms as Hybrid Products Comprising Mixtures of Antibodies, Antibody Fragments, and Small Molecule Inhibitors. These different modalities have different pharmacodynamics and pharmacokinetics, which may be suitable for neutralizing different families of venom toxins with distinct functions and toxicokinetics. Note that this schematic is all encompassing and that future hybrid products seem likely to contain a small number of the different modalities presented, rather than all of them simultaneously. Abbreviations: 3FTx, three-finger toxins; PLA2, phospholipases A2; SVMP, snake venom metalloproteinases; SVSP, snake venom serine proteases. Figure courtesy of: Tulika (Technical University of Denmark).

Ultimately, next-generation snakebite therapeutics may not necessarily be based on only one antitoxin format(e.g., antibodies or small molecule inhibitors), but instead seem likely to be composite products comprising mixtures of different modalities to ensure breadth of toxin neutralization across numerous distinct snakevenoms(Figure 3)[80,83,84]. The recent gains described earlier demonstrate that this is likely achievable in the future as long as sufficient knowledge about venom composition and variation is at hand. This further emphasizes the need for continued toxinological research into venom variation (see Outstanding Questions) and underlines the importance of bridging basic and applied sciences for the benefit of the world’s impoverished snakebite victims.

## Concluding Remarks

Toxin-encoding genes are members of some of the most dynamically evolving gene families found in nature and detailed studies of their molecular evolution can yield knowledge that is broadly applicable to the deepest questions in biology, particularly those concerning the origins of novel functions [2–5]. Whilst this evolutionary dynamism makes toxins an attractive research subject for molecular biologists, it has led to the creation of a pharmacologically diverse suite of toxic molecules that are the causative agents for the monumental clinical burden of snakebite envenoming observed today [10]. Venom variation, at both the inter- and intraspecific levels, results in diverse snakebite envenoming pathologies and presents a significant challenge to the development of broad-spectrum snakebite therapeutics [12–17]. Understanding the evolutionary processes generating this variation, and its functional and clinical consequences, is therefore of paramountimportance. However, we still lack a comprehensive understanding of the interplay between predator and prey ecology influencing the evolution of venom variation, and a current lack of genomic resources for snakes hamper our interpretations of the varying roles that different molecular mechanisms of gene evolution play in this regard (see Outstanding Questions). Nonetheless, progress is being made through an interdisciplinary research framework underpinned by other ‘omic’ technologies and combining perspectives and methods from evolutionary biology, immunology, and clinical toxinology. Current priorities for the application of this diverse data include robustly predicting the efficacy of existing antivenoms against untested snake species and identifying those toxins, found amongst numerous diverse isoforms present across all medically important snakes, that are of greatest importance to neutralize. Despite these challenges, recent research efforts are already beginning to yield valuable insights that are now being applied to the design and development of next-generation snakebite therapeutics. Particularly promising approaches include the utilization of monoclonal antibodies and repurposed small molecules that exhibit broad-spectrum neutralizing capacities against taxonomically widespread and clinically relevant toxin families, such as 3FTxs, dendrotoxins, PLA_2_s,and SVSPs [75,77–82]. Future broad-spectrum therapeutics will, thus, likely be developed by combining mixtures of these modalities in hybrid antivenom products. Much work remains to be done to strengthen our understanding of snake venoms as drug targets and to settle on the most optimal strategies for developing improved snakebite envenoming therapeutics, including identifying how many of these molecules are required to provide broad neutralization of diverse snake venoms [68,80,83,85]. However, recent achievements resulting from the mutually enlightening relationship between evolutionary and clinical toxinology provide away forward that may, in the near future, help save many thousands of lives and ease the burden of morbidity caused by snakebite envenoming in the developing tropical world.

## Highlights

Venom is an ecologically important functional trait in venomous snakes and its composition and activity often coevolve with the physiology of the prey animals it is deployed against.

Variation in toxin venom component soccurs both inter- and intraspecically as the result of various processes,including gene duplication and the action of positive selection.

The consequences of this variation is the generation of functionally diverse venoms that cause distinct pathologies in snakebite victims and which undermine the efficacy of antivenom treatments.

Knowledge of the varying toxins found across medically important venoms is enabling the generation of new therapeutic approaches to circumvent venom variation to better treat the worlds 1.8 million annual snakebite victims

## Box 1 Toxin Gene Family Expansion and Coevolution with Delivery Mechanisms

Venom composition varies as a consequence of contingent evolutionary history and direct functional selection. For example, specific families of toxin genes have undergone extensive expansion and neofunctionalization in specific snake lineages. Three-finger toxins (3FTxs) are a major component of the venom of elapid snakes and this gene family has expanded considerably via lineage-specific duplications, which have facilitated the emergence of multiple novel functions in addition to the ancestral activity of neurotoxic antagonism of nicotinic acetylcholine receptors [52,86]. Contrastingly, in viperid snakes, it is the snake venom metalloproteinases (SVMP) [17] and group II phospholipase A2 (PLA_2_) [38] toxin gene families that have undergone lineage-specific expansions, again facilitating the emergence of multiple novel activities. The multiple duplication events within these gene families result in redundant arrays of genes that form neofunctionalization hotspots [2,17,44,87,88]. In both the elapid and viperid snake lineages, gene expansions and rapid sequence diversifications have occurred following the evolution of front-fanged, high-pressure delivery systems, highlighting the revolutionary relationship that exists between toxin genes and venom delivery mechanisms [38,52].

## Glossary

Antivenom: conventional snakebite therapies that consist of polyclonal antibodies purified from plasma/serum of equines/ovines hyperimmunized with snake venom/s.
Aptamer: an oligonucleotide that binds to a specific target molecule. Cytotoxicity: the pathological consequences of venom cytotoxins acting predominantly at the region around the bite site, often resulting in swelling, blistering, and local tissue necrosis.
Elapidae: a widely distributed, medically important, family of front-fanged venomous snakes ('elapids') that often cause systemic neurotoxicity and that includes cobras, mambas, kraits, coral snakes, taipans, and relatives.
Hemotoxicity: the pathological consequences of venom hemotoxins acting on blood vessels and components of the coagulation cascade, often resulting in hypotension, systemic hemorrhage, and/or consumption coagulopathy.
Multilocus gene family: a gene family consisting of multiple copies that have arisen due to recurrent tandem duplications of the ancestral gene.
Nanobody: a single-domain antibody, typically of camelid origin, consisting of a single monomeric variable antibody domain (antibody fragment). Similar to a whole antibody, a nanobody can bind selectively to a specific antigen.
Neglected tropical diseases (NTDs): a diverse group of communicable and noncommunicable diseases that are common in low-income populations of Africa, Asia, and the Americas. Neofunctionalization: the acquisition of a new protein function as the result of an accumulation of mutations in duplicated genes.
Neurotoxicity: the pathological consequences of venom neurotoxins acting on neuromuscular junctions, often resulting in descending neuromuscular paralysis and respiratory failure.
Neutral evolution: in molecular evolutionary terms, this is a mode of evolution where genes accumulate (nearly) equal proportions of synonymous (changes in nucleotides that do not alter the encoded amino acid residue) and nonsynonymous (changes in nucleotides that alter the coded amino acid) substitutions.
Next-generation antivenom: future snakebite therapies that may consist of recombinantly expressed monoclonal antibodies, antibody fragments, alternative binding proteins, and/or small molecule inhibitors, as well as plasma/serum-derived antivenoms manufactured with the use of recombinant toxins (or fragments thereof) as immunogens.
Nonsynonymous substitutions: mutations in the nucleotides that change the encoded amino acid.
Peptide: a short chain of amino acids.
Positive selection: also known as positive Darwinian selection, it is the force of natural selection that favors the accumulation of nonsynonymous substitutions over synonymous substitutions and results in the diversification of gene sequences across evolutionary time.
Purifying selection: a force of natural selection thatfavors theaccumulationof synonymous substitutions over nonsynonymous substitutions, resulting in the conservation of gene sequences across evolutionary time.
Recombinant antivenom: antivenom based on recombinantly expressed antibodies or antibody fragments. Recombinant antivenoms can comprise either monoclonal, oligoclonal, or polyclonal antibodies.
Synonymous substitutions: changes at the nucleotide level that do not alter the resulting amino acid.
Venomvariation: relative differences in the composition and/or abundance of toxins in the venoms of closely/distantly related animals or their geographically disparate populations.
Viperidae: a widely distributed, medically important, family of frontfanged venomous snakes (‘vipers’) that often cause systemic hemotoxicity, and which includes pit vipers, vipers, adders, and relatives.

## Box 2 Timeworn Indian Antivenoms

Of the 300 species of Indian snakes described to date, 60 are capable of inflicting clinically significant envenomings in humans. However, the ‘big four’, the Indian cobra (*Naja* *naja*), Russell’s viper (*Daboia* *russelii*), common krait (*Bungarus* *caeruleus*), and saw-scaled viper (*Echis* *carinatus*), are responsible for the vast number of snakebite-related deaths and disabilities. While historically the Indian government estimated snakebite mortality to be low (e.g., 948 deaths in 2017), a comprehensive survey in 2011 projected that the actual burden of snakebite equated to 46 000 deaths and 140 000 cases of morbidity, making India the world’s snakebite hotspot [89].

To combat snakebite in India,polyvalent antivenom is produced by six antivenom manufacturers against the venoms of the ‘big four’ snakes. However, nearly all antivenom manufacturers source venom from a single geographical population of these snakes and thus there are grave concerns that intraspecific venom variation renders them less effective for treating bites in other parts of the country. Experimental evidence of this was recently demonstrated by an in vitro and in vivo evaluation of the efficacy of commercial antivenoms in countering the toxicities inflicted by north Indian population of B. *caeruleus* [16]. The tested commercial antivenom failed to meet the marketed neutralizing claim of antivenom potency, highlighting the negative impact of geographic venom variability on snakebite therapy.

In addition to these concerns, it is apparent that many other snake species are capable of inflicting human mortality and morbidity in India. These‘neglected many’are common in certain parts of the country that lack their ‘big four’ congeners, and include various species of cobra (*Naja kaouthia*, *Naja oxiana*), *kraits* (*Bungarus* *sindanus, Bungarus fasciatus*, *Bungarus niger*), and vipers (*Echis carinatus sochureki, Hypnale hypnale*). Despite being medically important, antivenoms are not manufactured against them and thus clinicians are forced to rely on therapeutics designed to neutralize venom of the ‘big four’ snakes. This is, however, despite venom characterization of the ‘neglected many’ revealing that their venom compositions and potencies are remarkably distinct [16]. In vitro and in vivo antivenom efficacy testing revealed that the existing Indian antivenoms have, at best, low efficacy against the venoms of the ‘neglected many’ and, at worst, fail to neutralize venoms from certain populations of some of these species [16]. The likely consequences of this are treatment failure [90] and the delivery of dangerously high volumes of ineffective, yet financially costly, antivenom to impoverished snakebite victims.

## Outstanding Questions

What are the relative contributions of ecological factors and functional evolution to the generation of snake venom variation?

What are the molecular mechanisms that generate venom variation and to what extent are these mechanisms described by prominent evolutionary models (e.g., ‘birth and death’ modelof gene evolution)?

How do we distinguish among highly related toxin isoforms those that are of the greatest importance to neutralize? How many toxins do we need to inhibit to prevent serious pathology in envenomed victims?

How feasible is it to develop a rational comparative framework to understand and predict whether existing antivenoms will neutralize the venoms of untested snakespecies?

How many novel antitoxin molecules are required to provide broad neutralizing breadth in the presence of extensive snake venom variation?

How does cost of manufacture influence the choice of optimal therapeutic intervention in different regions of the world?
